# Calcium-induced conformational changes in the regulatory domain of the human mitochondrial ATP-Mg/Pi carrier

**DOI:** 10.1016/j.bbabio.2015.07.002

**Published:** 2015-10

**Authors:** Steven P.D. Harborne, Jonathan J. Ruprecht, Edmund R.S. Kunji

**Affiliations:** The Medical Research Council, Mitochondrial Biology Unit, Cambridge Biomedical Campus, Wellcome Trust/MRC Building, Hills Road, Cambridge CB2 0XY, UK

**Keywords:** APC, ATP-Mg/Pi carrier, SCaMC, short calcium binding mitochondrial carrier, LMNG, lauryl maltose neopentyl glycol, DTT, dithiothreitol, EGTA, ethylene glycol tetraacetic acid, RMSD, root-mean-squared deviation, SEC, size exclusion chromatography, RD, regulatory domain, Calcium regulation mechanism, EF-hand conformational change, SCaMC, Adenine nucleotide translocase, Regulation of adenine nucleotides

## Abstract

The mitochondrial ATP-Mg/Pi carrier imports adenine nucleotides from the cytosol into the mitochondrial matrix and exports phosphate. The carrier is regulated by the concentration of cytosolic calcium, altering the size of the adenine nucleotide pool in the mitochondrial matrix in response to energetic demands. The protein consists of three domains; (i) the N-terminal regulatory domain, which is formed of two pairs of fused calcium-binding EF-hands, (ii) the C-terminal mitochondrial carrier domain, which is involved in transport, and (iii) a linker region with an amphipathic α-helix of unknown function. The mechanism by which calcium binding to the regulatory domain modulates substrate transport in the carrier domain has not been resolved. Here, we present two new crystal structures of the regulatory domain of the human isoform 1. Careful analysis by SEC confirmed that although the regulatory domain crystallised as dimers, full-length ATP-Mg/Pi carrier is monomeric. Therefore, the ATP-Mg/Pi carrier must have a different mechanism of calcium regulation than the architecturally related aspartate/glutamate carrier, which is dimeric. The structure showed that an amphipathic α-helix is bound to the regulatory domain in a hydrophobic cleft of EF-hand 3/4. Detailed bioinformatics analyses of different EF-hand states indicate that upon release of calcium, EF-hands close, meaning that the regulatory domain would release the amphipathic α-helix. We propose a mechanism for ATP-Mg/Pi carriers in which the amphipathic α-helix becomes mobile upon release of calcium and could block the transport of substrates across the mitochondrial inner membrane.

## Highlights

• The mechanism of calcium-regulation in the ATP-Mg/Pi carrier is unresolved.
• The human ATP-Mg/Pi carrier is monomeric in the presence and absence of calcium.
• In the presence of calcium an amphipathic α-helix binds in a cleft of EF-hand 3/4.
• Without calcium the amphipathic α-helix is released from the regulatory domain.
• Regulation could occur by the amphipathic α-helix blocking substrate translocation.

## Graphical abstract

**Figure:**
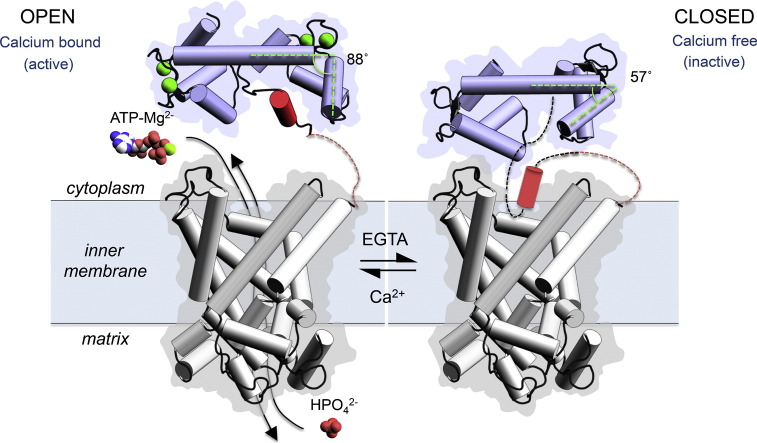

## 1 Introduction

The mitochondrial ATP-Mg/Pi carrier (APC) is a member of the mitochondrial carrier family of transport proteins. Mitochondrial carriers are typically located in the mitochondrial inner membrane and fulfil the vital role of shuttling nucleotides, amino acids, inorganic ions, keto acids, fatty acids and cofactors between the mitochondrial matrix and the cytosol [1]. APC is involved in the import of cytosolic adenine nucleotides into the mitochondrion and the export of inorganic phosphate from the mitochondrial matrix [2–4]. Mitochondria also have ADP/ATP carriers, a related but functionally distinct member of the mitochondrial carrier family [5]. In contrast to APC, ADP/ATP carriers catalyse the equimolar exchange of ADP from the cytosol for ATP synthesised in the mitochondrial matrix, meaning that their activity does not influence the size of the adenine nucleotide pool [5]. The unequal exchange of substrate catalysed by APC does have the potential to influence the total matrix adenine nucleotide pool. Therefore, APC has the important role of altering the mitochondrial adenine nucleotide pool in order to adapt to changing cellular energetic demands [6–8].

In yeast only one APC ortholog exists (*Sal1p*) [9], whereas in humans four genes encode full-length APC paralogs; *SLC25A24*, *SLC25A25*, *SLC25A23* and *SLC25A54* encoding for the protein short calcium binding mitochondrial carrier (SCaMC) isoform 1 (APC-1), SCaMC-2 (APC-3), SCaMC-3 (APC-2) and SCaMC-1L, respectively [3,10,11]. Another gene, *SLC25A41*, encodes for SCaMC-3L protein [12], which is shorter in sequence than the other APC isoforms (for a review see Satrústegui & Pardo, 2007) [13]. Mitochondrial carriers are fundamental to cellular metabolism, and have been implicated in many severe human diseases [14]. There is experimental support for the notion that up-regulation of human SCaMC-1 (HsAPC-1) expression in cancerous tissues may help these cells to evade cell death [15].

APC has a three-domain structure. The N-terminal domain of APC forms a calcium-sensitive regulatory domain [3,10]. The consequence of calcium binding to the regulatory domain of APC is a stimulation of the substrate transport activity of the carrier [2,4,7]. A structure of the regulatory domain of human APC isoform-1 has recently been solved [16], confirming the presence of four EF-hands, each of which is occupied by a bound calcium ion in a canonical pentagonal bi-pyramidal fashion. The EF-hands group together into two pairs, forming two lobes connected by a long central α-helix, in a similar fashion to the four EF-hands of calmodulin [16].

The C-terminal domain of APC is a membrane protein with the characteristic structural fold of mitochondrial carrier proteins [17]. The carrier fold is composed of three repeats [18], each containing two membrane-spanning α-helices connected by a matrix α-helix, making a three-fold pseudo-symmetrical protein structure [19,20]. Atomic structures of both bovine [19] and yeast ADP/ATP carriers [20] provide the basis for our understanding of the structure/function relationship between key sequence elements conserved across the carrier family. Based on the identification of a conserved central substrate binding site [21] flanked by two salt bridge networks on either side [22], an alternating access mechanism has been proposed for substrate transport through carrier proteins [20,22].

The linker region between the N-terminal regulatory domain and the C-terminal carrier domain contains an amphipathic α-helix of unknown function. In the structure this α-helix is bound to EF-hand pair 3 and 4, analogous to the binding of a calmodulin recognition sequence motif to the hydrophobic pockets in the EF-hands of calmodulin [16].

Another member of the carrier family of proteins, the aspartate/glutamate carrier (AGC), also displays a calcium-dependence for activity and has an N-terminal regulatory domain that contains EF-hands. Structures of both the calcium-bound and calcium-free states of the regulatory domain of the human AGC have recently been solved [23]. The structures reveal eight EF-hand motifs, only one of which is involved in calcium binding. Most mitochondrial carriers are monomeric [24], but AGC was found to be dimeric [23]. EF-hands four to eight are recruited to form an extensive interface for homo-dimerisation that is critical for the observed calcium-induced conformational changes within the regulatory domain of AGC [23]. Thus both APC and AGC have N-terminal regulatory domains consisting of EF-hands. Although no significant sequence similarity exists between these regulatory domains, there is the possibility that they share a common mechanism of calcium-regulation, potentially based on a homo-dimer arrangement. The protein construct used in the previous study of the HsAPC-1 regulatory domain was mutated at the extreme N-terminus to replace a cysteine residue at position 15 with a serine [16], raising the possibility that a native dimer could have been disrupted [25].

NMR nuclear Overhauser effects have shown that the regulatory domain of HsAPC-1 is more dynamic and less structured in the calcium-free state [16]. Binding studies with surface plasma resonance have indicated that the regulatory domain interacts with the carrier domain in the absence of calcium [16]. A mechanism was proposed in which the regulatory domain binds to the carrier domain in the absence of calcium, capping it in order to block substrate transport [16], however that mechanism does not explain why the bound calcium-free regulatory domain would be more dynamic. Therefore, the mechanism that couples calcium binding in the regulatory domain to substrate transport in the carrier domain has not been resolved.

Here, an independent structural determination of the native human APC-1 regulatory domain was completed, which was combined with an investigation into the oligomeric state of the full-length human APC-1 protein. Furthermore, an extensive comparison of the conformations of EF-hand proteins in the calcium-free, calcium-bound and peptide-bound state was undertaken, which has allowed us to propose a mechanism for the regulation of APC that is consistent with all available experimental data.

## 2 Materials and methods

### 2.1 Expression and purification of full-length HsAPC-1

A construct of HsAPC-1 (Uniprot: Q6NUK1) was designed to include an eight-histidine tag followed by a Factor Xa cleavage site (IEGR) at the N-terminus, creating a cleavable purification-tag. The gene was codon-optimized for expression in *S. cerevisiae* by GenScript and cloned into a pYES3/CT expression plasmid (Invitrogen), with the inducible galactose promoter being replaced by the constitutive promoter for the yeast mitochondrial phosphate carrier, as described previously [26]. The expression plasmid was transformed into *S. cerevisiae* haploid strain W303-1b [27], using established methods [28].

*S. cerevisiae* was cultured, and HsAPC-1 was expressed following established methods [20], with the following modifications: pre-cultures of yeast were set up in synthetic-complete tryptophan-dropout medium (Formedium) supplemented with 2% glucose, and the main cultures were carried out in an Applikon bioreactor with 100 L YEPD medium. Mitochondria were prepared using established methods [29], flash frozen in liquid nitrogen, and stored under liquid nitrogen until use.

HsAPC-1 was solubilised in lauryl maltose neopentyl glycol (LMNG), and separated from insoluble protein by ultracentrifugation. Solubilised protein was passed through nickel sepharose affinity resin (GE Healthcare) to selectively bind full-length HsAPC-1, and washed with 50 column volumes of buffer A (20 mM Tris pH 7.4, 150 mM NaCl, 20 mM Imidazole, 0.1% (w/v) LMNG, 0.1 mg mL^− 1^ tetra-oleoyl cardiolipin) and 30 column volumes of buffer B (20 mM Tris pH 7.4, 50 mM NaCl, 0.1% (w/v) LMNG, 0.1 mg mL^− 1^ tetra-oleoyl cardiolipin). Factor Xa protease (New England BioLabs) was used to specifically cleave the HsAPC-1 protein from the affinity resin. After Factor Xa cleavage, HsAPC-1 was spun through an empty Proteus midi-spin column (Generon) to remove affinity resin. Protein concentrations were determined using the bicinchoninic acid assay [30] against a bovine serum albumin standard curve (Pierce). Purified protein was used immediately for analysis using SEC.

### 2.2 Expression and purification of HsAPC-1 regulatory domain

Primers were design in order to create a construct of HsAPC-1 to include just the regulatory domain and the linker region (residues 14 to 174) of the protein (Forward primer: GAAGGTAGAACCTCCGAAGA. Reverse primer: CCTAGGTCTAGACTCGAGTCATTATTATATATCAATACCTGTGGAATGTTT). The inter-domain loop was not included in this construct. The construct was cloned into the *Lactococcus lactis* expression plasmid pNZ8048, and the plasmid transformed into electrocompetent *L. lactis* NZ9000 using established methods [31]. Expression of the construct was carried out as described previously [23]. Purification of HsAPC-1 was achieved using a three-step chromatography protocol. First, clarified *L. lactis* lysate was passed over Ni sepharose resin (GE Healthcare). The resin was washed with 50 column volumes of purification buffer (20 mM Tris pH 7.5, 150 mM NaCl, 1 mM DTT) containing 50 mM imidazole and eluted in purification buffer containing 250 mM imidazole. Second, recovered protein was concentrated, and desalted using a PD10 column (GE Healthcare). The purification tag was removed from the construct by digestion with Factor Xa protease (Supplementary Fig. 1). Finally protein was loaded onto a Superdex 200 pg 16/60 SEC column (GE Healthcare) in a running buffer of 20 mM Tris pH 7.5, 20 mM NaCl. Peak fractions were collected and concentrated to approximately 10 mg mL^− 1^ for crystallography.

### 2.3 Experimental analysis of oligomeric state

Superdex 200 pg 16/60 SEC columns (GE Healthcare) were calibrated with molecular weight standards from both the high and low molecular weight standards kits (GE Healthcare), and unknown protein weights were estimated using the manufacturers recommended protocol. The SEC buffer used for analysis of the HsAPC-1 RD protein was purification buffer with either 5 mM calcium or 5 mM EGTA, with or without 1 mM DTT added as stated. The SEC buffer used for analysis of the full-length HsAPC-1 was 20 mM Tris pH 7.4, 50 mM NaCl, 0.03% (w/v) LMNG and 0.03 mg mL^− 1^ tetra-oleoyl cardiolipin. The buffers used to run the molecular weight standards in each case matched the buffers being used in the analysis of each protein.

Total detergent, phospholipid and protein contributions to the total mass could be determined colorimetrically by sugar assay [32], phosphorus assay [33] and bicinchoninic acid assay [30], respectively. SDS-PAGE gels for the analysis of full-length HsAPC-1 were composed of 12% acrylamide and run using a Tris–Glycine buffer system. SDS-PAGE gels for the analysis of HsAPC-1 RD were composed of 10% acrylamide and run using a Tris–Tricine buffer system. Gels were stained with Imperial coomassie stain (Bio-Rad), and de-stained in water.

### 2.4 HsAPC-1 regulatory domain crystallisation, data collection and structural determination

Crystallisation screening with purified HsAPC-1 RD yielded an initial hit in drop H4 of the JBS Classics screen (Jena Bioscience) with a mother-liquor composed of 30% (w/v) polyethylene glycol 8000, 0.2 M ammonium sulfate. Optimisation around this hit produced square plate-like crystals in 29% (w/v) polyethylene glycol 8000, 0.2 M ammonium sulfate, larger in both width and height than the original hit, approximately 200 × 200 μm in dimension.

Crystals of a tetrahedral bi-pyramidal morphology could be grown in 25–30% (w/v) polyethylene glycol 8000, 0.2 M lithium sulfate conditions. These crystals were smaller than the square plate-like crystals, with dimensions of about 40 × 30 × 30 μm, and grew as well defined single crystals.

High-resolution diffraction datasets were collected at Diamond beamline I24 and ESRF beamline ID23-2 for the *P*2 and *P*2_1_2_1_2_1_ crystals, respectively. A long-wavelength dataset of the *P*2_1_2_1_2_1_ crystals used for a calcium-SAD experiment was also collected at Diamond beamline I24.

Using the known HsAPC-1 RD structure as a molecular replacement template [16] the structure of the HsAPC-1 RD was solved in two different space groups: to 2.1 Å resolution in space-group *P*2 from the square plate-like crystals, and to 2.6 Å in the space-group *P*2_1_2_1_2_1_ from the tetrahedral bi-pyramidal type crystals. Refinement statistics are shown in (Table 1). High-resolution datasets were integrated using *iMOSFLM* [34], followed by scaling and merging with *POINTLESS* and *AIMLESS* [35] in the CCP4 suite [36]. The long-wavelength dataset was processed by the pipeline Xia2 [36] using 3D spot integration. For molecular replacement phenix.phaser within the *PHENIX* suite [37] of software was used. After initial placement of molecular replacement models, 50 rounds of jelly body refinement in *REFMAC5* [36] were used to improve phases before rounds of manual building and refinement were undertaken. Final rounds of refinement were carried out using phenix.refine in the *PHENIX* suite [37] of software including the use of Translation/Libration/Screw groups, selected using phenix.find_TLS_groups.

The long-wavelength dataset was phased by molecular replacement with the refined *P*2_1_2_1_2_1_ model using phenix.phaser. The anomalous difference map was calculated using phenix.find_peaks_and_holes.

Phasing by molecular replacement of the *P*2 dataset was originally only successful in P1 as the space-group was initially assigned as *P*2_1_. The program *ZANUDA* in the CCP4 suite [36] was used to identify the correct higher symmetry space-group as *P*2. For manual building of the model into density the program *COOT* was used [38].

### 2.5 Structural interpretation and representation

All structures were visualised and diagrams prepared using PyMOL (version 1.7.0.2) [39]. The program PISA in the CCP4 suite [36] was used to evaluate the dimerisation interfaces of dimeric proteins in Table 3. The solvent filled cavity was visualised using the surface view mode in PyMOL [39] set to ‘cavities and pockets’, with surface cull set to ‘47’, surface detection radius set to 5 solvent radii and surface cut-off radius set to 4 solvent radii.

### 2.6 Comparison of EF-hand structures

Uniprot was used to search for structures of EF-hand proteins with a Pfam [40] assignment of EF-hand 7 or EF-hand 8, which both indicate pairs of EF-hands. Each EF-hand was superposed on EF-hand 1 of calcium-bound calmodulin using 10 residues of the entering α-helix to position 5 of the EF-hand loop. In total 129 EF-hands were aligned with one another (Supplementary Table 1). A line was drawn through the centre of both the entering and exiting α-helix, and the angle between the lines calculated. A histogram of all the angles was plotted using Prism 5.0 (GraphPad) and a Gaussian distribution of the angles each subset of EF-hands exhibit, were calculated.

### 2.7 Modelling calcium-free HsAPC-1

We propose α-helix 4/5 represents a static element connecting the two lobes of the protein together. The pitch of the α-helix 4/5 allowed precise manual alignment [39] of the calcium-free EF-hand pair models independently of the position of the other α-helices of the calcium-bound lobes of the HsAPC-1 RD structure. This process was repeated, replacing both lobe 1 and 2 with each respective reference model. Residues from the end of α-helix 8 onwards were excluded from the models, as no reference for the position of the amphipathic α-helix in the calcium-free state is available. The MODELLER [41] web-server was then used to perform comparative protein structure modelling in order to replace the residues of the reference structures with those of HsAPC-1 RD. This approach included the use of spatial restraints and CHARMM energy terms in order to preserve the proper stereochemistry, and an optimisation step using the variable target function method [42].

### 2.8 Author contributions

S.P.D.H. expressed, purified and crystallised proteins, performed biochemical assays and carried out the structure determination. All authors were involved in experimental planning, data collection at synchrotrons, data analysis and manuscript preparation.

### 2.9 Accession numbers

The coordinates and structure factors of the calcium-bound regulatory domain of the ATP-Mg/Pi carrier in both the *P*2 and *P*2_1_2_1_2_1_ forms have been deposited in the Protein Data Bank under accession codes PDB ID: 4ZCU and PDB ID: 4ZCV, respectively.

## 3 Results and discussion

### 3.1 The HsAPC-1 regulatory domain structure

A construct of the regulatory domain (RD) and the linker region (residues 14 to 174) of HsAPC-1 was expressed in *L. lactis* and purified. HsAPC-1 RD was crystallised using sitting drop vapour diffusion techniques, predominantly in two different conditions. Molecular replacement using the recently solved structure of the HsAPC-1 RD (PDB ID: 4N5X) [16], yielded solutions, which were refined to produce models with good R-factors and geometry statistics in space-groups *P*2_1_2_1_2_1_ or *P*2 (Table 1).

**Fig. 1:**
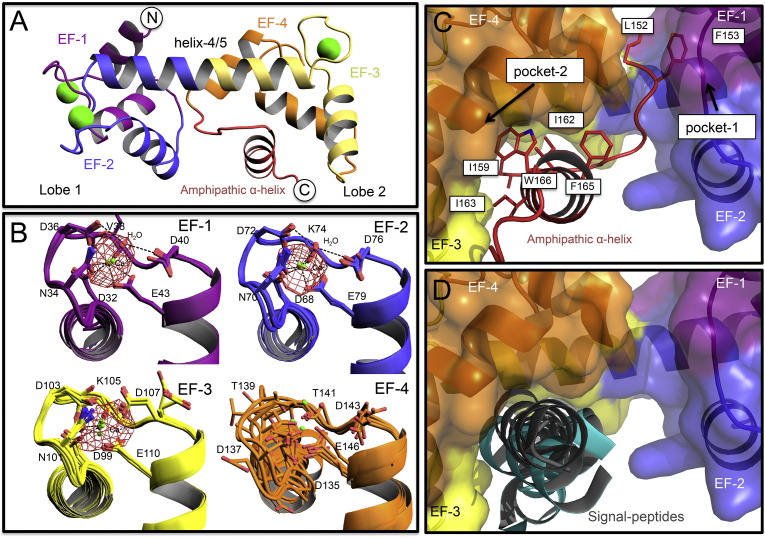
Features of the HsAPC-1 RD structure. A) A single chain of the HsAPC-1 regulatory domain (RD) structure. B) Detailed views of the EF-hands where each observation in the seven chains is superposed. Electron density from an anomalous difference Fourier map, calculated from the *P*2_1_2_1_2_1_ space-group, is displayed as a mesh in red (σ-level of 5). C) View of hydrophobic pockets 1 and 2 that bind the loop preceding the amphipathic α-helix and the amphipathic α-helix itself, respectively, or D) the position of calmodulin recognition sequence motifs (black for calmodulin peptides or cyan for others) when EF-hands of other proteins are superposed on lobe 2 of the HsAPC-1 RD structure. In panels A and B a cartoon representation of the HsAPC-1 RD molecule is shown, but in panels C and D a surface representation is used. In all panels EF-hands 1 to 4 are coloured in purple, blue, yellow and orange respectively. The amphipathic α-helix is represented in red. Calcium ions are represented as lime green spheres, and red spheres represent water molecules. In all cases, where side chains are shown, they are represented as sticks, and nitrogen and oxygen atoms are coloured according to convention.

As was found previously [16], the HsAPC-1 RD structure is formed of two lobes connected by a long α-helix (helix 4/5) (Fig. 1A), with each lobe composed of a pair of EF-hands. Furthermore, each EF-hand displays an ‘open’ antiparallel arrangement of α-helices (including EF-hand 4), equivalent to that of calcium-bound calmodulin structures [43–47].

For each chain, electron density was observed for residues 23 to 174. For four of the chains there was a break in electron density between residues 139 and 141 (EF-hand 4), and calcium could only be modelled into EF-hand 4 in the three chains of the *P*2 space group, but not the four chains in the *P*2_1_2_1_2_1_ space-group (Fig. 1B). This is in contrast with the previously solved structure, which showed calcium binding in all four EF-hands [16].

It was previously suggested that an interaction between the terminal amphipathic α-helix and the hydrophobic pocket of HsAPC-1 RD might be a crystallisation artefact [16]. Here we independently confirm this interaction (Fig. 1C), even though the expression, purification and crystallisation procedures, and the crystal packing was different. It was previously noted that the interaction of the amphipathic α-helix with pocket-2 mimics the binding of signal-peptides into the hydrophobic pockets in calmodulin [16]. This analysis was extended (Table 2) by superimposing structures of other α-helix binding EF-hand pairs onto lobe 2 of HsAPC-1 RD independently of the bound α-helix. The bound α-helix in each case matched the position of the amphipathic α-helix well (Fig. 1D), with α-helices bound to calmodulin being most similar in position (Table 2). We conclude that this interaction is not an artefact, and propose that the amphipathic α-helix has a specific role in the regulatory mechanism.

One unusual feature of the interaction between the hydrophobic pocket and its target, the amphipathic α-helix, is that they are both part of the same polypeptide chain. Other EF-hand proteins, such as calmodulin, interact with target α-helices of other proteins. However, the APC regulatory domain is not unique in this type of ‘self-sequestered’ interaction. Other examples are Kv channel interacting protein-1 [48], Guanylate activating protein 1 [49], AGC [23], stroma interacting molecule-1 [50], and Calcyphosin [51].

The interaction between regulatory domain and the amphipathic α-helix resembles that of AGC and the C-terminal α-helix, and given that they are both members of the mitochondrial carrier family of proteins it is tempting to conclude that they might have an analogous mechanism. However, a fundamental difference is that in AGC the carrier domain is inserted between the EF-hands and the target α-helix, whereas in APC the target α-helix is inserted between the EF-hands and the carrier domain.

### 3.2 Oligomeric state of HsAPC-1

**Fig. 2:**
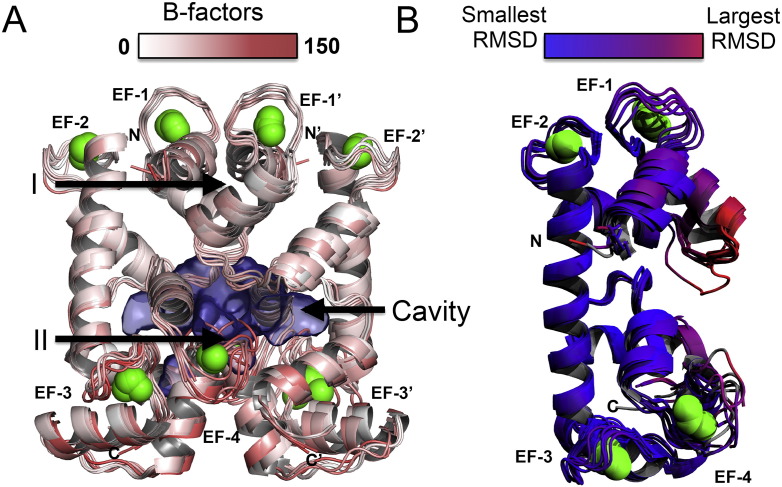
Crystal packing of HsAPC-1 RD chains, and comparison to previous HsAPC-1 RD structure. A) Seven unique dimer combinations of HsAPC-1 RD in total were superposed upon one another. Each dimer is represented as a cartoon and coloured according to B-factor values as indicated in the key. The two main points of contact between each chain of the dimer are labelled I and II respectively. B) The seven observed chains of the HsAPC-1 RD were superposed on the previously published one (PDB ID: 4N5X). The structures in panel B are coloured by the RMSD difference from 4N5X as in the key. Calcium ions are represented as lime green spheres.

In both crystal space-groups, HsAPC-1 RD was crystallised as a parallel homo-dimer (Fig. 2A). In the previous study, it was crystallised as a monomer, but Cys15 was replaced by serine [16]. The dimer interface is composed of the α-helices of the EF-hands (helices 1, 2, 3, 6, and 7), and is focussed at two contact points, one point from each EF-hand pair. All of the HsAPC-1 RD chains observed here differ slightly from the previous HsAPC-1 RD structure (PDB ID: 4N5X) around α-helices 2 and 3 (Fig. 2B). These α-helices are involved in the first contact point of the dimerisation interface, which could explain these differences.

In total, between the two space groups, seven copies of the HsAPC-1 RD were observed in this study. Each chain has a solvent accessible surface area of approximately 8800 Å [36], of which 1400 Å [36] is buried by the dimer interface. All seven copies can be superposed upon one another with good agreement: RMSD 0.552 Å (± 0.270 Å) over all atoms in each chain. Furthermore, the dimers can also be superposed with good agreement: RMSD 0.629 Å (± 0.475 Å) over all atoms in each dimer. Thus the configuration of the dimers is very similar between the two space groups, even though they are packed very differently.

One of the contact points of dimerisation involves the calcium-binding loop of EF-hand 4, a site that had notably high B-factors and poor electron density (Fig. 2A). Between the two contact points there is a large solvent-filled cavity. In comparison to structures of proteins with proven dimerisation interfaces, the two chains of HsAPC-1 bury a comparable surface area, however the theoretical solvation energy gain was more comparable to that of recoverin or neurocalcin that have an interface for transient dimerisation (Table 3). The interface between HsAPC-1 protomers contains relatively few hydrogen bonds and no salt-bridge interactions, which is more comparable to the crystal contacts observed between chains of calmodulin or calcyphosin (Table 3).

**Fig. 3:**
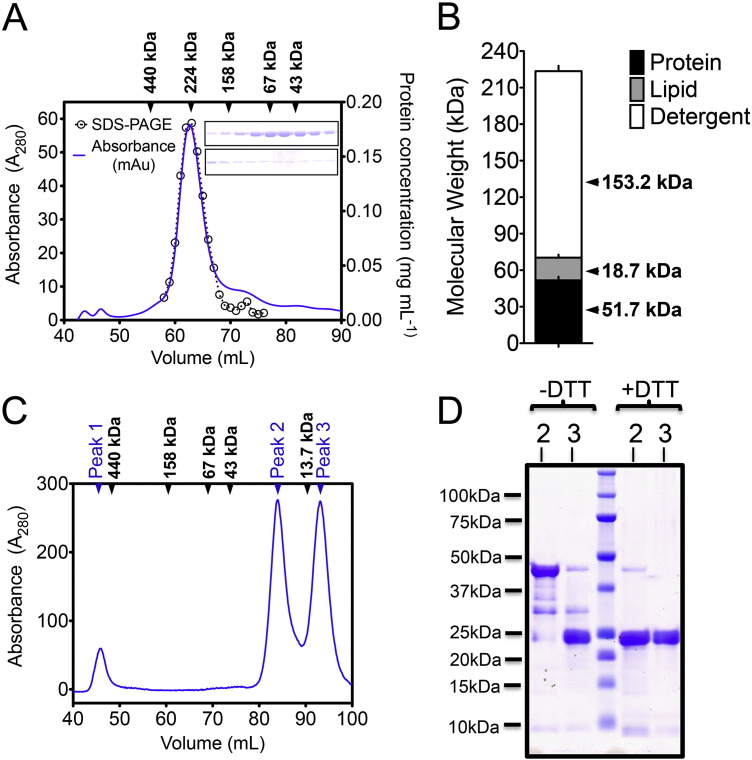
Full-length HsAPC-1 is monomeric. A) SEC trace for full-length HsAPC-1 and protein quantification by SDS-PAGE gel (inset). B) Contributions of protein, detergent and lipid to the total mass. C) SEC trace for crudely isolated HsAPC-1 RD. Peak 1 was found to contain a contaminant, and identified as the 56.1 kDa E2 component of the *L. lactis* pyruvate dehydrogenase complex (Supplementary Fig. 1). In panels A and C the elution volume of molecular weight standards are indicated and annotated with the mass of the standard. D) Protein from each of the peaks 2 and 3 run on an SDS-PAGE gel in the presence or absence of DTT.

To clarify this issue further, an independent experimental analysis of the oligomeric state of the HsAPC-1 was conducted, first on the full-length HsAPC-1 (Fig. 3). The protein was expressed in yeast, solubilised in LMNG, purified and analysed by size exclusion chromatography (SEC) (Fig. 3A). Compared to molecular weight standards, the total mass of the HsAPC-1 protein:detergent:lipid micelle was 223.5 kDa. An experimental assessment of the individual contributions to the total mass revealed that 153.2 kDa could be attributed to detergent, 18.7 kDa could be attributed to lipid, with the remaining mass, 51.2 kDa, being attributed to protein. Full-length HsAPC-1 has a theoretical molecular mass of 53.4 kDa, demonstrating that the protein is monomeric in detergent (Fig. 3B).

Next the oligomeric state of the isolated HsAPC-1 RD construct was investigated. During purification, two dominant peaks with retention volumes of 85 mL and 94.5 mL were observed in SEC traces (peak 2 and peak 3) (Fig. 3C). By SDS-PAGE analysis combined with peptide mass fingerprinting, both peaks 2 and 3 were found to contain HsAPC-1 RD (Supplementary Fig. 1). The theoretical mass of the HsAPC-1 RD protein construct is 21 kDa. The estimated molecular weight of the protein in peak 2 (56 kDa) corresponds to the theoretical mass of a regulatory domain dimer, and peak 3 (28 kDa) is consistent with the mass of a monomer (Supplementary Fig. 2).

It was important to determine whether the observed dimerisation of the isolated RD could be influenced by calcium. Fractions from peak 2 and peak 3 were pooled separately, and analysed by SEC in the presence of calcium or ethylene glycol tetraacetic acid (EGTA), and with or without the reducing agent dithiothreitol (DTT). The elution volumes of the dimer and monomer species were both affected (Table 4 and Supplementary Fig. 2), but the shift in estimated size between the two conditions was not enough to account for the difference between a monomer and a dimer.

The addition of the reducing agent DTT caused the protein in peak 2 to shift in size closer to the mass expected for the monomeric species in both calcium and EGTA conditions (Table 4 and Supplementary Fig. 2). Therefore, dimerisation appears to be caused by disulphide bond formation between proteins (Fig. 3D), and is not influenced by calcium binding (Table 4 and Supplementary Fig. 2). The cysteine residue in the HsAPC-1 RD sequence (Cys15) that is involved in the disulphide bond is not conserved in other APC orthologs (Supplementary Fig. 3).

To conclude; (i) the dimerisation interface between the two chains of HsAPC-1 does not conform to expected characteristics for a physiologically relevant dimer, (ii) the cysteine involved in covalent dimerisation is not conserved and (iii) no effect of calcium on disulphide bond formation could be observed. Therefore, APC is monomeric and dimers of the HsAPC-1 RD observed in SEC traces and in crystal structures appear to be caused by non-specific disulphide bond formation between Cys15 residues and by crystal packing, respectively. Thus the mechanism of calcium regulation of transport must be different from that of AGC, which is dimeric.

### 3.3 Modelling conformational changes in the HsAPC-1 regulatory domain

The HsAPC-1 RD has been solved in a calcium-bound state. However, to appreciate the mechanism by which the regulatory domain would influence substrate transport, the calcium-free state must also be investigated. Even though attempts have been made here and previously [16], it was not possible to obtain crystal hits in the presence of EGTA. Instead structures of other EF-hand proteins within the PDB were studied to derive the most likely conformational change in the absence of calcium.

A diverse reference set of EF-hand containing proteins was selected from the PDB with a preference for those containing EF-hands organised in pairs. Both crystal structures and NMR models of calcium-bound and calcium-free states were selected (Supplementary Table 1). In total, over 100 EF-hands were structurally superposed.

**Fig. 4:**
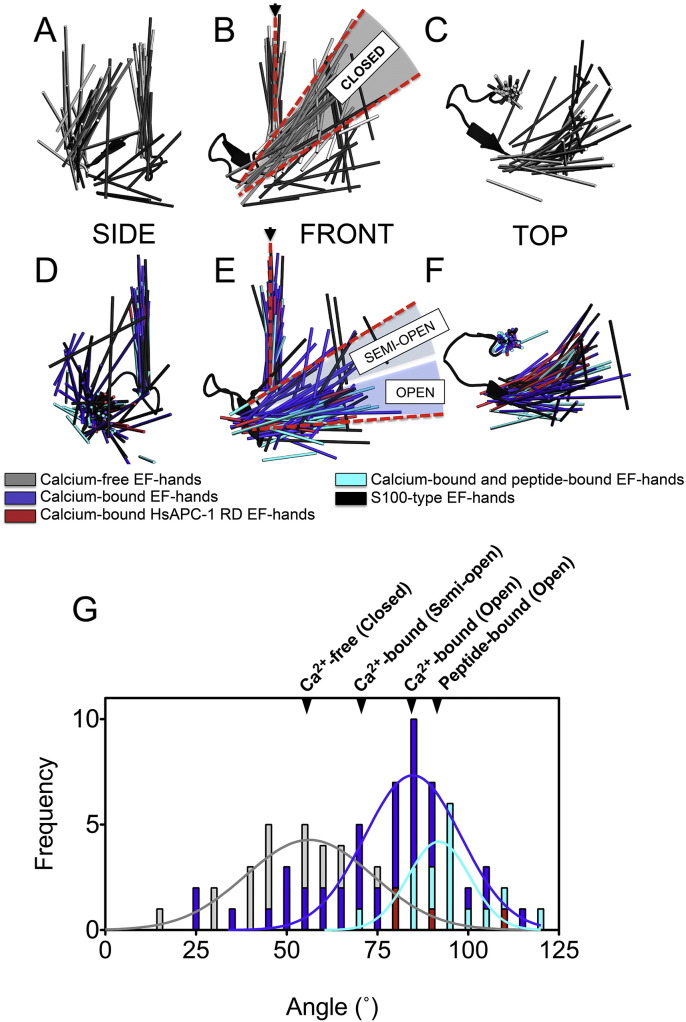
Calcium-induced conformational changes in EF-hands. Views of 129 EF-hands superposed on EF-hand 1 of calcium-bound calmodulin, in the calcium-free (A–C) and calcium-bound (D–F) states. Entering and exiting α-helices are represented as a line though the α-helix centre. G) A histogram of the angles between the entering and exiting α-helices of conventional (not S100-like) EF-hands.

Calcium-bound EF-hands displayed open or semi-open conformations. The angle of the exiting α-helix relative to the entering α-helix was 70° to 90° and 55° to 70°, respectively (Fig. 4). Calcium-free EF-hands displayed generally a closed conformation (45° to 65°). These results agree with a previous study [52], but expand the reference set to include a larger number of EF-hands published since the previous study was conducted.

The largest variations were seen for the S100-like EF-hands, which have an additional two residues in the calcium-binding loop, and therefore provide a wider variety of possible conformations of the exiting α-helix. Some of the EF-hands within AGC are S100-like, but all four EF-hands of HsAPC-1 are conventional EF-hands, and adopt the fully open conformation as expected for calcium-bound EF-hands (Fig. 4 and Supplementary Table 1).

In order to understand the conformational changes that occur when HsAPC-1 goes from the calcium-bound to the calcium-free state, models of the calcium-free state were generated based on deposited structures. Three models of known calcium-free EF-hand pairs (with conventional EF-hand motifs) were chosen as reference structures for modelling; centrin (PDB ID: 1ZMZ) with an angle of ~ 40°, N-terminal lobe of calmodulin (PDB ID: 1CFD) with an angle of ~ 55° and the N-terminal lobe of calcium-dependent protein kinase (PDB ID: 3HZT) with an angle of ~ 65°.

**Fig. 5:**
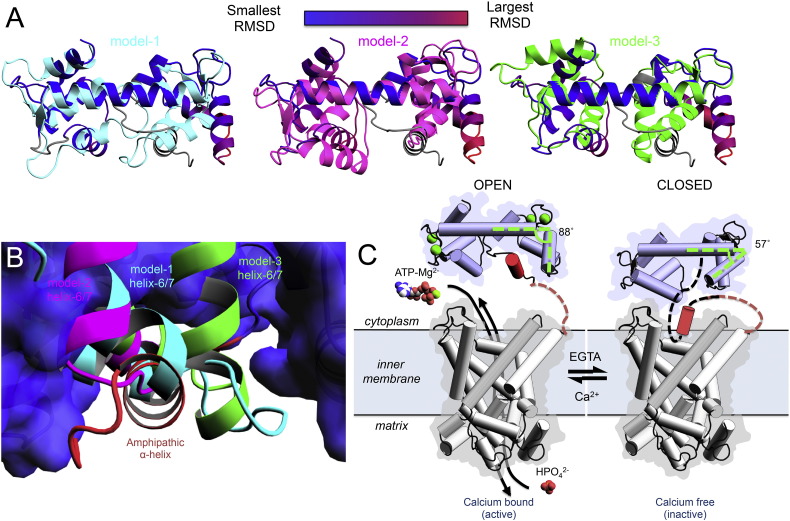
A model for the calcium regulation of HsAPC-1 RD. A) Three models for calcium-free HsAPC-1 RD based on centrin (model-1), calmodulin (model-2) and calcium-dependent protein kinase (model-3) were superposed onto the calcium-bound HsAPC-1 RD structure and coloured cyan, magenta and green respectively. The calcium-bound HsAPC-1 structure is coloured by RMSD difference from the calcium-free models as in the key. B) A close up view of hydrophobic pocket 2 into which the amphipathic α-helix (red) is bound. The calcium-bound HsAPC-1 RD structure is shown as a surface representation and coloured blue, whereas the superposed calcium-free models are represented as cartoon (coloured as in panel A). C) The proposed regulatory mechanism between calcium-bound state of HsAPC-1 where the carrier is active and the calcium-free state of HsAPC-1 where the carrier is inactive. The calcium-free model presented in panel C is based on model 2. α-helices are represented as cylinders in panel C. Calcium ions are presented as lime green spheres.

Although the three resulting models had some structural differences from one another, the general motion that they described was consistent (Fig. 5A). All calcium-free models show that α-helices 6 and 7 adopt a position that would prevent the binding of the amphipathic α-helix to hydrophobic pocket 2 (Fig. 5B). Therefore, in the absence of calcium, the hydrophobic pockets of HsAPC-1 RD close, and as a consequence the amphipathic α-helix is excluded (Supplementary video 1). All three models agree with this mechanism, but model 2 is preferred, as it represents the median of calcium-free EF-hand structures (Fig. 4G). In support of this mechanism, the isolated APC regulatory domain becomes more dynamic in the absence of calcium, particularly in the region of the amphipathic α-helix as demonstrated by nuclear Overhauser effect experiments [16]. Furthermore, we demonstrate here using SEC that under calcium-free condition the hydrodynamic radius of the isolated APC regulatory domain is increased in comparison with the calcium-bound state (Table 4), consistent with the exclusion of the amphipathic α-helix from the pocket.

It is possible that when the amphipathic α-helix is released, it could interact with the carrier domain to inhibit substrate transport, either by blocking access to the binding site or by preventing conformational changes required for transport by the carrier domain (Fig. 5D). This model for the mechanism has more consistent features with an EF-hand mechanism than the “capping and uncapping” mechanism, previously proposed [16].

## 4 Conclusion

The evidence presented here indicates that the functional unit of APC, including the isolated regulatory domain, is a monomer. Therefore, APC differs from AGC, which has a regulatory mechanism based on conformational changes within a dimeric regulatory domain. Thus they must be different in the key functional elements involved in regulation. Importantly, any mechanism for calcium regulation of transport must be based on APC functioning as a monomer.

So far only structures of the calcium-bound state have been solved (Fig. 1) [16], but it was possible to propose a model for the calcium-free state based on known structures of calcium-free EF-hand pairs (Fig. 5). We propose a conformational change in the HsAPC-1 RD where the amphipathic α-helix fulfils a role similar to that of a calmodulin recognition sequence motif, even though it is part of the same protein. We propose that upon the removal of calcium, the EF-hands close, leading to displacement of the amphipathic α-helix from the hydrophobic pocket. Once released, the amphipathic α-helix would be in an ideal position to interact with the carrier domain and block transport of substrates.

The following are the supplementary data related to this article.

Supplementary material

Supplementary video 1 Animation of the calcium-induced changes in HsAPC-1 RD.
Morph-model between the calcium-bound HsAPC-1 RD crystal structure and model-2 for the calcium-free HsAPC-1 RD. Structures displayed and coloured as in Fig. 1.

## Transparency document

Transparency document.

## Figures and Tables

**Table 1:** HsAPC-1 regulatory domain structure refinement statistics.

Space group	*P*2_1_2_1_2_1_ 4ZCV	*P*2 4ZCU	*P*2_1_2_1_2_1_–Ca^2 +^-SAD^c^
Wavelength (Å)	1.002	0.873	1.823
Resolution range (Å)	40.10–2.80 (2.95–2.80)	39.39–2.1 (2.16–2.10)	40.40–4.35 (4.505–4.35)
Unit cell	75.92 77.01 119.90 90 90 90	76.08 47.02 93.24 90 108.5 90	375.76 77.63 121.17 90 90 90
Total reflections	57586	118930	165332
Unique reflections	17779	36885	5031
Multiplicity	3.2 (3.2)	3.2 (3.2)	32.9 (27.5)
Completeness (%)	99.6 (99.7)	99.67 (100.00)	99.7 (98.3)
Anomalous multiplicity	–	–	17.9 (17.4)
Anomalous completeness (%)	–	–	99.8 (98.3)
Anomalous signal^a^	–	–	0.1304
Mean I/sigma(I)	8.4 (2.8)	7.25 (1.47)	17.90 (5.94)
Wilson B-factor	51.03	27.62	57.776
R-merge (%)	9.8 (40.0)	11.9 (95.8)	23.8 (102.8)
R-meas (%)	12.2 (53.7)	14.3 (115.3)	24.8 (105.9)
R-work	0.2110	0.2364	
R-free	0.2590	0.2745	
Number of non-hydrogen atoms	4859	3922	
Water molecules	50	168	
Protein residues	599	454	
Number of molecules per ASU^b^	4	3	
RMS(bonds)	0.011	0.0096	
RMS(angles)	0.927	0.829	
Ramachandran favoured (%)	97.5	97.5	
Ramachandran outliers (%)	0	0.5	
Clash score	8.66	9.09	
Average B-factor	64	50	

Statistics for the highest-resolution shell are shown in parentheses.

a Anomalous signal calculated by phenix.Xtriage.

b Asymmetric unit (ASU).

c Single wavelength anomalous dispersion (SAD).

**Table 2:** Superposition of signal peptides with the amphipathic α-helix.

Protein	Peptide bound	PDB	Residues^a^	EF-hands RMSD (Å)^b^	Peptide RMSD (Å)^c^	Reference
Calmodulin	Rs20	1QS7	5–74	0.753	2.780	Unpublished
Calmodulin	MLCK	2BBN	5–74	1.510	5.094	[53]
Calmodulin	PMCA R-domain	4AQR	5–74	1.113	9.631	[54]
Troponin C	Troponin I	3TZ1	83–149	3.112	9.301	[55]
KCNIP1	Self^d^	1S1E	123–170	0.163	11.403	[48]
GCAP1	Self^d^	2R2I	86–129	0.419	9.665	[49]
Citrin^e^	Self^d^	4P5W	12–87	0.959	12.062	[23]

Abbreviations: Myosin light chain kinase (MLCK), Calcium-transporting ATPase 8, Plasma membrane type (PMCA), Kv channel interacting protein-1 (KCNIP1), Guanylate activating protein 1 (GCAP1).

a EF-hands were aligned to residues 88–151 of the HsAPC-1 RD structure.

b RMSD calculated using the align function in PyMOL (version 1.7.0.2) and represents the value after outlier rejection.

c RMSD calculated using rms_cur function in PyMOL (version 1.7.0.2) and represents the value without superposition and without rejecting outliers.

d The α-helix with which the EF-hands are interacting is part of the same polypeptide chain.

e Synonym for citrin is AGC-2.

**Table 3:** Comparison of dimerisation interfaces in EF-hand containing proteins.

Protein	PDB code	Monomer or dimer in solution	Surface of monomer (Å^2^)	Surface area buried by dimerization			hydrogen bonds	salt-bridges	Solvation energy gain (kcal mol^− 1^)
				(Å^2^)		(%)			
HsAPC-1 RD	4ZCV	–	8716	1394		16	4	0	− 16.6
Calmodulin	1CLL	Monomer	9721.0	612.4		6	2	4	1.0
Calcyphosin	3E3R	Monomer	11376.8	824.5		7	7	0	− 7.5
Recoverin	1REC	Monomer/dimer	9565	1060		11	9	1	− 13.9
Neurocalcin	1BJF	Monomer/dimer	10554	1161		11	8	4	− 9.5
AGC RD	4P5W	Dimer	15719	2242		14	15	2	− 22.0
S100A15	4AQI	Dimer	6248	1136		18	13	0	− 21.3
Calpain	1DVI	Dimer	10781	2085		19	20	1	− 21.8

Crystal contacts 2–5% in all cases.

**Table 4:** Oligomeric state of HsAPC-1 RD under different conditions.

Original oligomeric state	Conditions of second SEC column			Elution volume (mL)	Estimated molecular weight (kDa)	Oligomeric state after second SEC column
Ca^2 +^	EGTA	DTT
Monomer	✓			93.8	25	Monomer
Dimer	✓			84.8	51	Dimer
Dimer	✓		✓	93.8	25	Monomer
Monomer		✓		90.3	34	Monomer
Dimer		✓		79.3	80	Dimer
Dimer		✓	✓	90.3	34	Monomer
