# Supplementary material for: Calcium-induced conformational changes in the regulatory domain of the human mitochondrial ATP-Mg/Pi carrier
Source: Biochim Biophys Acta. 2015 Oct;1847(10):1245–53. doi: 10.1016/j.bbabio.2015.07.002 (PMC4562336; doi:10.1016/j.bbabio.2015.07.002)
Supplement: Supplementary file 1 — Supplementary material [file mmc1.docx]

**Supplementary Fig. 1**

##
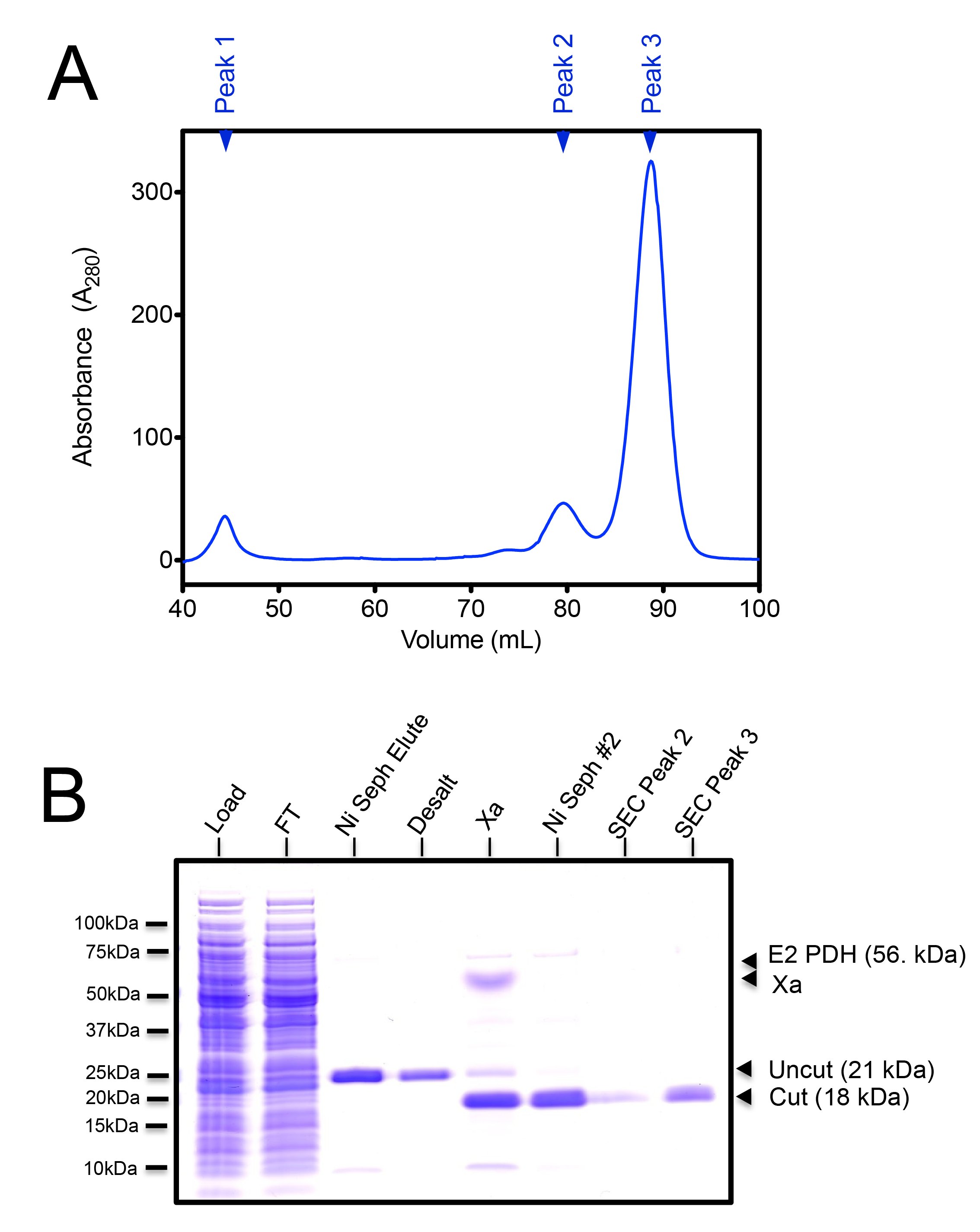


**Supplementary Fig. 2**

##
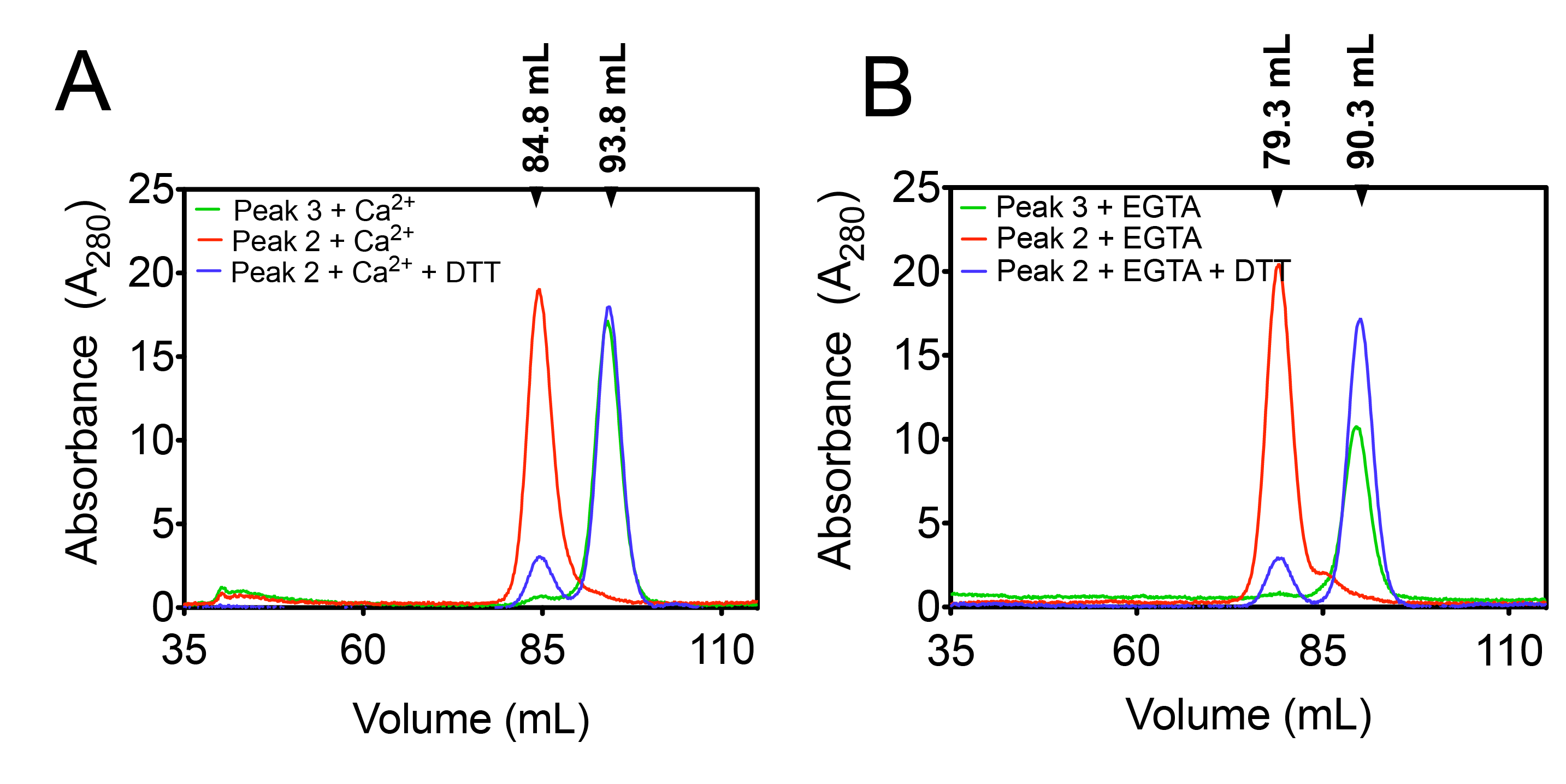


**Supplementary Fig. 3**

**
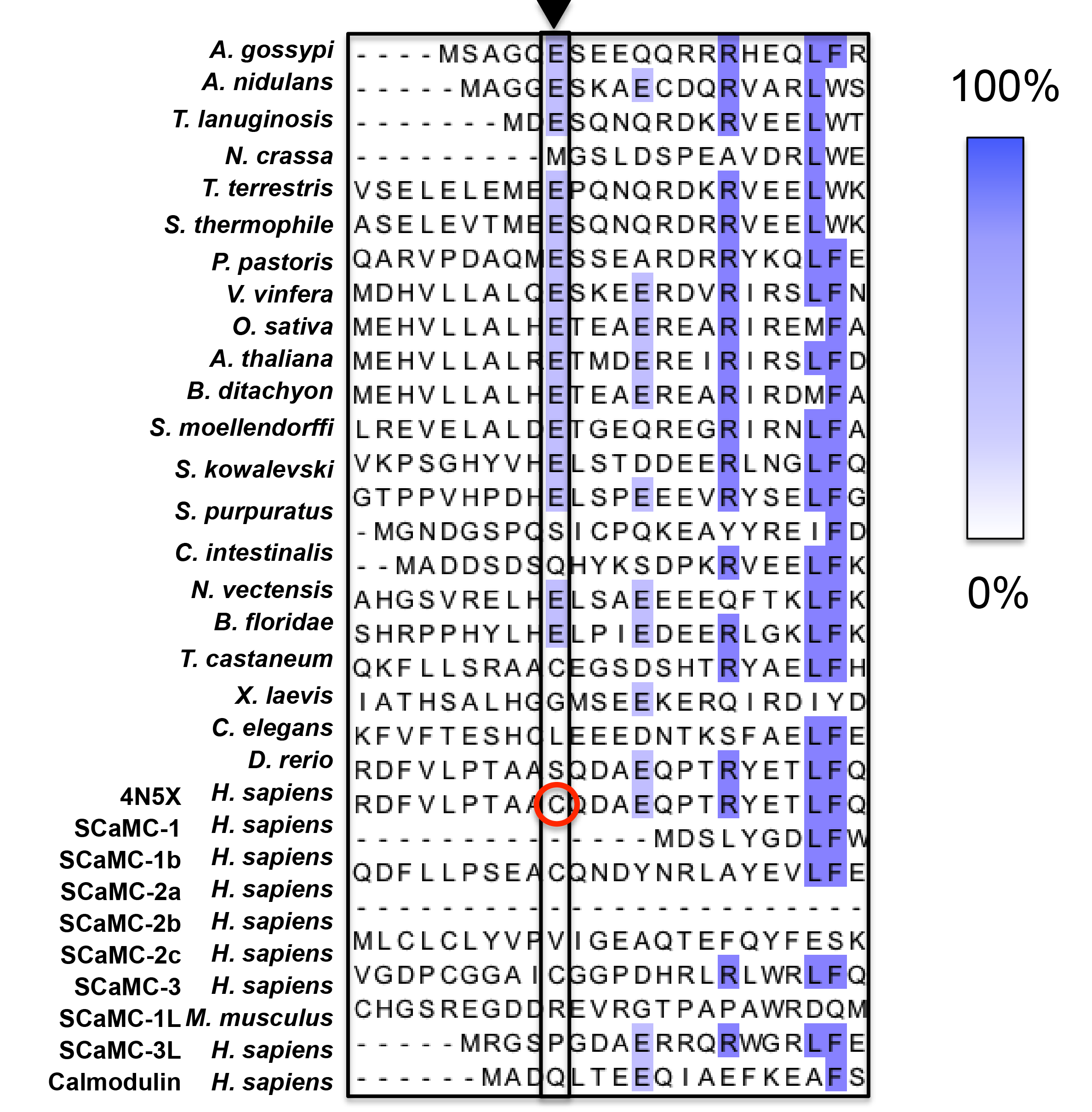
**

## Supplemental Figure Legends

**Supplementary Fig. 1 –** Purification of HsAPC-1 RD

A) The elution of protein from a Superdex pg 200 16/60 column by monitoring the UV absorbance at 280 nm (A280) as a function of buffer volume (blue trace). Sample was incubated with 1 mM DTT before passage down the column. Three protein peaks were observed, and have been labelled. B) Samples throughout the course of HsAPC-1 protein purification (using the second protocol for purification) were taken and loaded into a 10% acrylamide Tris-tricine SDS-PAGE gel, stained with Imperial stain. The identity and theoretical molecular weight of the proteins in different bands are indicated. Abbreviations: flow through (FT), Factor Xa (Xa), nickel sepharose (Ni Seph), size exclusion chromatography (SEC), pyruvate dehydrogenase (PDH).

**Supplementary Fig. 2** - SEC Traces of HsAPC-1 RD

SEC traces of HsAPC-1 RD in the A) presence of calcium or B) EGTA. Green traces indicate HsAPC-1 RD pooled from peak 2 of the initial SEC column. Red traces indicate HsAPC-1 RD pooled from peak 3 of the initial SEC column. Blue traces indicate HsAPC-1 RD pooled from peak 3 of the initial SEC column run in the presence of DTT.

**Supplementary Fig. 3 -** C15S of HsAPC-1 RD is not conserved

An alignment of the N-terminus of APC orthologs reveals that C15S of HsAPC-1 RD is not conserved. Residues are coloured according to the percentage of the residues in each column that agree with the consensus sequence as in the key. Only the residues that agree with the consensus residue for each column are coloured. Alignment and images of the alignment were prepared with Jalview.^1^

**Supplementary Table 1.**

| **Protein** | **PDB code** | **EF hand** | **Divalent cation** | **Angle (˚)^a^** | **Peptide bound** | **Method** | **Resolution (Å)** | **Organism** | **Date** | **Reference** |
| --- | --- | --- | --- | --- | --- | --- | --- | --- | --- | --- |
| **SCaMC-1**  **(APC-1)** | 4ZCV | 1 | Ca^2+^ | 75.38 | helix-9 | X-ray | 2.1 | *Homo sapiens* | 2015 | here |
|  |  | 2 | Ca^2+^ | 76.95 |  |  |  |  |  |  |
|  |  | 3 | Ca^2+^ | 88.08 |  |  |  |  |  |  |
|  |  | 4 | None | 109.6 |  |  |  |  |  |  |
|  | 4N5X | 1 | Ca^2+^ | 80.21 | helix-9 | X-ray | 2.1 | *Homo sapiens* | 2014 | 2 |
|  |  | 2 | Ca^2+^ | 75.67 |  |  |  |  |  |  |
|  |  | 3 | Ca^2+^ | 89.77 |  |  |  |  |  |  |
|  |  | 4 | Ca^2+^ | 103.69 |  |  |  |  |  |  |
| **Calcyphosin** | 3E3R | 1 | Ca^2+^ | 82.06 | helix-1 | X-ray | 2.65 | *Homo sapiens* | 2008 | 3 |
|  |  | 2 | Ca^2+^ | 78.71 |  |  |  |  |  |  |
|  |  | 3 | Ca^2+^ | 101.63 |  |  |  |  |  |  |
|  |  | 4 | Ca^2+^ | 122.39 |  |  |  |  |  |  |
| **Aralar**  **(AGC-1)** | 4P5X | 1 | - | 68.32 | - | X-ray | 2.26 | *Homo sapiens* | 2014 | 4 |
|  |  | 2 | Ca^2+^ | 99.92 |  |  |  |  |  |  |
|  |  | 3 | - | 90.97 |  |  |  |  |  |  |
|  |  | 4 | - | 5.02 |  |  |  |  |  |  |
|  | 4P60 | 2 | None | 106.19 | - | X-ray | 2.4 | *Homo sapiens* | 2014 | 4 |
|  |  | 3 | None | 91.7 |  |  |  |  |  |  |
|  |  | 4 | None | 11.96 |  |  |  |  |  |  |
| **Calmodulin** | 1CFD | 1 | None | 62.71 | - | NMR | N/A | *Xenopus laevis* | 1995 | 5 |
|  |  | 2 | None | 61.7 |  |  |  |  |  |  |
|  |  | 3 | None | 51.93 |  |  |  |  |  |  |
|  |  | 4 | None | 47.52 |  |  |  |  |  |  |
|  | 1CLL | 1 | Ca^2+^ | 79.86 | - | X-ray | 1.7 | *Homo sapiens* | 1992 | 6 |
|  |  | 2 | Ca^2+^ | 86.42 |  |  |  |  |  |  |
|  |  | 3 | Ca^2+^ | 85.8 |  |  |  |  |  |  |
|  |  | 4 | Ca^2+^ | 81.37 |  |  |  |  |  |  |
|  | 1VRK | 1 | Ca^2+^ | 98.81 | Rs20 | X-ray | 1.9 | *Gallus gallus* | 1999 | 7 |
|  |  | 2 | Ca^2+^ | 107.11 |  |  |  |  |  |  |
|  |  | 3 | Ca^2+^ | 94.08 |  |  |  |  |  |  |
|  |  | 4 | Ca^2+^ | 89.13 |  |  |  |  |  |  |
| **Caltractin/ Centrin 2** | 1ZMZ | 1 | None | 39.48 | - | NMR | N/A | *Homo sapiens* | 2006 | 8 |
|  |  | 2 | None | 44.69 |  |  |  |  |  |  |
|  | 2AMI | 1 | Ca^2+^ | 68.31 | - | NMR | N/A | *Chlamydomonas reinhardtii* | 2005 | 9 |
|  |  | 2 | Ca^2+^ | 82.29 |  |  |  |  |  |  |
| **Troponin C (N-terminal domain)** | 1AVS | 1 | Ca^2+^ | 94.43 | - | NMR | N/A | *Gallus gallus* | 1997 | 10 |
|  |  | 2 | Ca^2+^ | 91.8 |  |  |  |  |  |  |
| **Troponin C (C-terminal domain)** | 2K2A | 3 | None | 58.41 | - | NMR | N/A | *Lethocerus indicus* | 2010 | 11 |
|  |  | 4 | None | 50.7 |  |  |  |  |  |  |
|  | 3TZ1 | 3 | Ca^2+^ | 88.25 | Troponin I | NMR | N/A | *Chlamys nipponensis akazara* | 2012 | 12 |
|  |  | 4 | Ca^2+^ | 77.72 |  |  |  |  |  |  |
| **Troponin C**  **(full-length)** | 1TOP | 1 | None | 35.5 | - | X-ray | 1.78 | *Gallus gallus* | 1994 | 13 |
|  |  | 2 | None | 39.25 |  |  |  |  |  |  |
|  |  | 3 | Ca^2+^ | 83.49 |  |  |  |  |  |  |
|  |  | 4 | Ca^2+^ | 71.98 |  |  |  |  |  |  |
| **ALG2 (Programmed cell death protein 6)** | 2ZN9 | 1 | Ca^2+^ | 86.08 | - | X-ray | 2.4 | *Homo sapiens* | 2008 | 14 |
|  |  | 2 | - | 56.5 |  |  |  |  |  |  |
|  |  | 3 | Ca^2+^ | 68.46 |  |  |  |  |  |  |
|  |  | 4 | - | 68.15 |  |  |  |  |  |  |
|  |  | 5 | Ca^2+^ | 24.17 |  |  |  |  |  |  |
| **Sorcin** | 1JUO | 1 | None | 49.42 | - | X-ray | 2.2 | *Homo sapiens* | 2001 | 15 |
|  |  | 2 | None | 78.32 |  |  |  |  |  |  |
|  |  | 3 | None | 55.49 |  |  |  |  |  |  |
|  |  | 4 | None | 50.85 |  |  |  |  |  |  |
|  |  | 5 | None | 40.92 |  |  |  |  |  |  |

**Supplementary Table 1 cont.**

| **Calpain** | 1AJ5 | 1 | None | 44.67 | - | X-ray | 2.3 | *Rattus norvegicus* | 1997 | 16 |
| --- | --- | --- | --- | --- | --- | --- | --- | --- | --- | --- |
|  |  | 2 | None | 65.83 |  |  |  |  |  |  |
|  |  | 3 | None | 41.09 |  |  |  |  |  |  |
|  |  | 4 | None | 52.44 |  |  |  |  |  |  |
|  |  | 5 | None | 40.61 |  |  |  |  |  |  |
|  | 1DV1 | 1 | Ca^2+^ | 52.01 | - | X-ray | 2.3 | *Rattus norvegicus* | 1997 | 16 |
|  |  | 2 | Ca^2+^ | 69.23 |  |  |  |  |  |  |
|  |  | 3 | Ca^2+^ | 53.3 |  |  |  |  |  |  |
|  |  | 4 | - | 59.1 |  |  |  |  |  |  |
|  |  | 5 | - | 34.43 |  |  |  |  |  |  |
| **Calcium dependent protein kinase** | 3HX4 | 1 | Ca^2+^ | 85.59 |  | X-ray | 1.95 | *Toxoplasma gondii* | 2010 | 17 |
|  |  | 2 | Ca^2+^ | 112.12 |  |  |  |  |  |  |
|  |  | 3 | Ca^2+^ | 99.72 |  |  |  |  |  |  |
|  |  | 4 | Ca^2+^ | 92.6 |  |  |  |  |  |  |
|  | 3HZT | 1 | None | 60.59 |  | X-ray | 2.0 | *Toxoplasma gondii* | 2010 | 17 |
|  |  | 2 | None | 79.97 |  |  |  |  |  |  |
|  |  | 3 | None | 74.09 |  |  |  |  |  |  |
| **Calcyclin (S100A6)** | 1CNP | 1 | Ca^2+^ | 81.63 | - | NMR | N/A | *Oryctolagus cuniculus* | 1995 | 18 |
|  |  | 2 | Ca^2+^ | 45.53 |  |  |  |  |  |  |
|  | 1A03 | 1 | None | 56.48 | - | NMR | N/A | *Oryctolagus cuniculus* | 1998 | 19 |
|  |  | 2 | None | 32.58 |  |  |  |  |  |  |
| **S100B** | 1MHO | 1 | Ca^2+^ | 61.11 | - | X-ray | 2.0 | *Bos taurus* | 1998 | 20 |
|  |  | 2 | Ca^2+^ | 82.17 |  |  |  |  |  |  |
|  | 1CFP | 1 | None | 52.48 | - | NMR | N/A | *Bos taurus* | 1996 | 21 |
|  |  | 2 | None | 27.68 |  |  |  |  |  |  |
| **S100A16** | 2L51 | 1 | Ca^2+^ | 40.16 | - | NMR | N/A | *Homo sapiens* | 2010 | 22 |
|  |  | 2 | Ca^2+^ | 22.42 |  |  |  |  |  |  |
|  | 2L50 | 1 | None | 62.06 | - | NMR | N/A | *Homo sapiens* | 2010 | 22 |
|  |  | 2 | None | 26.81 |  |  |  |  |  |  |
| **Recoverin** | 1REC | 1 | Ca^2+^ | 61.67 | - | X-ray | 1.9 | *Bos taurus* | 1993 | 23 |
|  |  | 2 | Ca^2+^ | 72.46 |  |  |  |  |  |  |
|  |  | 3 | Ca^2+^ | 102.17 |  |  |  |  |  |  |
|  |  | 4 | Ca^2+^ | 83.5 |  |  |  |  |  |  |
|  | 1IKU | 1 | None | 23.94 | - | NMR | N/A | *Bos taurus* | 1995 | 24 |
|  |  | 2 | None | 56.69 |  |  |  |  |  |  |
|  |  | 3 | None | 73.94 |  |  |  |  |  |  |
|  |  | 4 | None | 67.71 |  |  |  |  |  |  |
| **Calbindin D9k** | 2BCB | 1 | Ca^2+^ | 49.85 | - | NMR | N/A | *Bos taurus* | 1993 | 25 |
|  |  | 2 | Ca^2+^ | 77.32 |  |  |  |  |  |  |
|  | 1CLB | 1 | None | 70.04 | - | NMR | N/A | *Bos taurus* | 1995 | 26 |
|  |  | 2 | None | 67.3 |  |  |  |  |  |  |
| **Parvalbumin (oncomodulin)** | 2NLN | 1 | None | 114.94 | - | NMR | N/A | *Rattus norvegicus* | 2007 | 27 |
|  |  | 2 | None | 108.98 |  |  |  |  |  |  |
|  | 1B8R | 1 | Ca^2+^ | 83.3 | - | X-ray | 1.9 | *Cyprinus carpio* | 1999 | 28 |
|  |  | 2 | Ca^2+^ | 81.55 |  |  |  |  |  |  |
| **MCFD-2** | 2VRG | 1 | Ca^2+^ | 48.69 | - | NMR | N/A | *Homo sapiens* | 2008 | 29 |
|  |  | 2 | Ca^2+^ | 91.52 |  |  |  |  |  |  |
| **Calsenilin/Kv interacting protein 4** | 1S1E | 1 | - | 75.88 | helix-10 | X-ray | 2.3 | *Homo sapiens* | 2004 | 30 |
|  |  | 2 | - | 83.61 |  |  |  |  |  |  |
|  |  | 3 | Ca^2+^ | 91.03 |  |  |  |  |  |  |
|  |  | 4 | Ca^2+^ | 85.85 |  |  |  |  |  |  |
| **Guanylyl cyclase-activating protein 1** | 2R2I | 1 | - | 66.66 | helix-10-myristoyl | X-ray | 2.0 | *Gallus gallus* | 2007 | 31 |
|  |  | 2 | Ca^2+^ | 82.56 |  |  |  |  |  |  |
|  |  | 3 | Ca^2+^ | 93.14 |  |  |  |  |  |  |
|  |  | 4 | Ca^2+^ | 94.35 |  |  |  |  |  |  |
| **STIM1** | 2K60 | 1 | Ca^2+^ | 94.92 | helix-10 | NMR | N/A | *Homo sapiens* | 2008 | 32 |
|  |  | 2 | - | 115.64 |  |  |  |  |  |  |
| **FKPB 14** | 4MSP | 1 | Ca^2+^ | 91.76 | EF 1-2 loop | X-ray | 1.9 | *Homo sapiens* | 2014 | 33 |
|  |  | 2 | Ca^2+^ | 105.95 |  |  |  |  |  |  |
| ‘-‘ indicates where calcium was present, however the calcium ion was not observed in that particular EF-hand  ^a^Angles between helices were calculated using the PyMOL script getanglesbetweenhelices.py  Abbreviations: Multiple coagulation factor deficiency 2 (MCFD-2) | | | | | | | | | | |

### Supplementary References

1. Waterhouse, A.M., Procter, J.B., Martin, D.M.A., Clamp, M., and Barton, G.J. (2009). Jalview Version 2-a multiple sequence alignment editor and analysis workbench. Bioinformatics, 25, 1189–1191.
2. Yang, Q., Brüschweiler, S., and Chou, J.J. (2014). A Self-Sequestered Calmodulin-like Ca²⁺ sensor of mitochondrial SCaMC carrier and its implication to Ca²⁺-dependent ATP-Mg/P(i) transport. Structure, 22, 207-217
3. Dong, H., Li, X., Lou, Z., Xu, X., Su, D., Zhou, X., Zhou, W., Bartlam, M., and Rao, Z. (2008). Crystal-Structure and Biochemical Characterization of Recombinant Human Calcyphosine Delineates a Novel EF-hand-Containing Protein Family. J. Mol. Biol., 383, 455–464.
4. Thangaratnarajah, C., Ruprecht, J.J., and Kunji, E.R.S. (2014). Calcium-induced conformational changes of the regulatory domain of human mitochondrial aspartate/glutamate carriers. Nature Comms., 5, 1–12.
5. Kuboniwa, H., Tjandra, N., Grzesiek, S., Ren, H., Klee, C.B., and Bax, A. (1995). Solution structure of calcium-free calmodulin. Nat. Struct. Biol., 2, 768–776.
6. Chattopadhyaya, R., Meador, W.E., Means, A.R., and Quiocho, F.A. (1992). Calmodulin structure refined at 1.7 Å resolution. J. Mol. Biol., 228, 1177–1192.
7. Mirzoeva, S., Weigand, S., Lukas, T.J., Shuvalova, L., Anderson, W.F., and Watterson, D.M. (1999). Analysis of the functional coupling between calmodulin's calcium binding and peptide recognition properties. Biochemistry, 38, 3936–3947.
8. Yang, A., Miron, S., Duchambon, P., Assairi, L., Blouquit, Y., and Craescu, C.T. (2006). The N-terminal domain of human centrin 2 has a closed structure, binds calcium with a very low affinity, and plays a role in the protein self-assembly. Biochemistry, 45, 880–889.
9. Sheehan, J.H. (2005). Structure of the N-terminal calcium sensor domain of centrin reveals the biochemical basis for domain-specific function. J. Biol. Chem., 281, 2876–2881.
10. Strynadka, N.C.J., Cherney, M., Sielecki, A.R., Li, M.X., Smillie, L.B., and James, M.N.G. (1997). Structural details of a calcium-induced molecular switch: X-ray crystallographic analysis of the calcium-saturated N-terminal domain of troponin C at 1.75 Å resolution. J. Mol. Biol., 273, 238–255.
11. De Nicola, G.F., Martin, S., Bullard, B., and Pastore, A. (2010). Solution structure of the apo C-Terminal domain of the lethocerusF1 Troponin C Isoform. Biochemistry, 49, 1719–1726.
12. Kato, Y.S., Yumoto, F., Tanaka, H., Miyakawa, T., Miyauchi, Y., Takeshita, D., Sawano, Y., Ojima, T., Ohtsuki, I., and Tanokura, M. (2013). Structure of the Ca^2+^-saturated C-terminal domain of scallop troponin C in complex with a troponin I fragment. Biol. Chem., 394, 55–68.
13. Satyshur, K.A., Pyzalska, D., Greaser, M., Rao, S.T., and Sundaralingam, M. (1994). Structure of chicken skeletal muscle troponin C at 1.78 A resolution. Acta Crystallogr. D, 50, 40–49.
14. Suzuki, H., Kawasaki, M., Inuzuka, T., Okumura, M., Kakiuchi, T., Shibata, H., Wakatsuki, S., and Maki, M. (2008). Structural basis for Ca^2+^-Dependent formation of ALG-2/Alix peptide complex: Ca^2+^/EF3-driven arginine switch mechanism. Structure, 16, 1562–1573.
15. Xie, X., Dwyer, M.D., Swenson, L., Parker, M.H., and Botfield, M.C. (2001). Crystal structure of calcium-free human sorcin: a member of the penta-EF-hand protein family. Prot. Sci., 10, 2419–2425.
16. Blanchard, H., Grochulski, P., Li, Y., Arthur, J.S., Davies, P.L., Elce, J.S., and Cygler, M. (1997). Structure of a calpain Ca^2+^-binding domain reveals a novel EF-hand and Ca^2+^-induced conformational changes. Nat. Struct. Biol., 4, 532–538.
17. Wernimont, A.K., Artz, J.D., Finerty, P., Lin, Y.-H., Amani, M., Allali-Hassani, A., Senisterra, G., Vedadi, M., Tempel, W., Mackenzie, F., Chau, I., Lourido, S., Sibley, L.D., and Hui, R. (2010). Structures of apicomplexan calcium-dependent protein kinases reveal mechanism of activation by calcium. Nat. Struct. Mol. Biol., 17, 596–601.
18. Potts, B.C., Smith, J., Akke, M., Macke, T.J., Okazaki, K., Hidaka, H., Case, D.A., and Chazin, W.J. (1995). The structure of calcyclin reveals a novel homodimeric fold for S100 Ca^2+^-binding proteins. Nat. Struct. Biol., 2, 790–796.
19. Sastry, M., Ketchem, R.R., Crescenzi, O., Weber, C., Lubienski, M.J., Hidaka, H., and Chazin, W.J. (1998). The three-dimensional structure of Ca^2+^-bound calcyclin: implications for Ca^2+^-signal transduction by S100 proteins. Structure, 6, 223–231.
20. Matsumura, H., Shiba, T., Inoue, T., Harada, S., and Kai, Y. (1998). A novel mode of target recognition suggested by the 2.0 Å structure of holo S100B from bovine brain. Structure, 6, 233–241.
21. Kilby, P.M., Van Eldik, L.J., and Roberts, G.C. (1996). The solution structure of the bovine S100B protein dimer in the calcium-free state. Structure, 4, 1041–1052.
22. Babini, E., Bertini, I., Borsi, V., Calderone, V., Hu, X., Luchinat, C., and Parigi, G. (2010). Structural characterization of human S100A16, a low-affinity calcium binder. J Biol. Inorg. Chem., 16, 243–256.
23. Flaherty, K.M., Zozulya, S., Stryer, L., and McKay, D.B. (1993). Three-dimensional structure of recoverin, a calcium sensor in vision. Cell, 75, 709–716.
24. Tanaka, T., Amest, J.B., Harvey, T.S., and Stryer, L. (1995). Sequestration of the membrane-targeting myristoyl group of recoverin in the calcium-free state. Nature, 376, 444-447.
25. Kördel, J., Skelton, N.J., Akke, M., and Chazin, W.J. (1993). High-resolution Solution Structure of calcium-loaded calbindin D 9k. J. Mol. Biol. 231, 711–734.
26. Skelton, N.J., Kördel, J., and Chazin, W.J. (1995). Determination of the solution structure of Apo calbindin D9k by NMR spectroscopy. J. Mol. Biol. 249, 441–462.
27. Henzl, M.T., and Tanner, J.J. (2007). Solution structure of Ca^2+^-free rat β-parvalbumin (oncomodulin). Prot. Sci., 16, 1914–1926.
28. Cates, M.S., Berry, M.B., Ho, E.L., Li, Q., Potter, J.D., and Phillips, G.N. (1999). Metal-ion affinity and specificity in EF-hand proteins: coordination geometry and domain plasticity in parvalbumin. Structure, 7, 1269–1278.
29. Guy, J.E., Wigren, E., Svärd, M., Härd, T., and Lindqvist, Y. (2008). New Insights into multiple coagulation factor deficiency from the solution structure of human MCFD2. J. Mol. Biol. 381, 941–955.
30. Scannevin, R.H., Wang, K., Jow, F., Megules, J., Kopsco, D.C., Edris, W., Carroll, K.C., Lü, Q., Xu, W., Xu, Z., Katz, A.H., Olland, S., Lin, L., Taylor, M., Stahl, M., Malakian, K., Somers, W., Mosyak, L., Bowlby, M.R., Chanda, P., and Rhodes, K.J. (2004). Two N-terminal domains of Kv4 K^+^ channels regulate binding to and modulation by KChIP1. Neuron, 41, 587–598.
31. Stephen, R., Bereta, G., Golczak, M., Palczewski, K., and Sousa, M.C. (2007). Stabilizing function for myristoyl group revealed by the crystal structure of a neuronal calcium sensor, guanylate cyclase-activating protein 1. Structure, 15, 1392–1402.
32. Stathopulos, P.B., Zheng, L., Li, G.-Y., Plevin, M.J., and Ikura, M. (2008). Structural and mechanistic insights into STIM1-mediated initiation of store-operated calcium entry. Cell, 135, 110–122.
33. Boudko, S.P., Ishikawa, Y., Nix, J., Chapman, M.S., and Bächinger, H.P. (2013). Structure of human peptidyl-prolyl cis-transisomerase FKBP22 containing two EF-hand motifs. Prot. Sci., 23, 67–75.
