# Supplementary figures and images for: Calcium-induced conformational changes in the regulatory domain of the human mitochondrial ATP-Mg/Pi carrier
Source: Biochim Biophys Acta. 2015 Oct;1847(10):1245–53. doi: 10.1016/j.bbabio.2015.07.002 (PMC4562336; doi:10.1016/j.bbabio.2015.07.002)

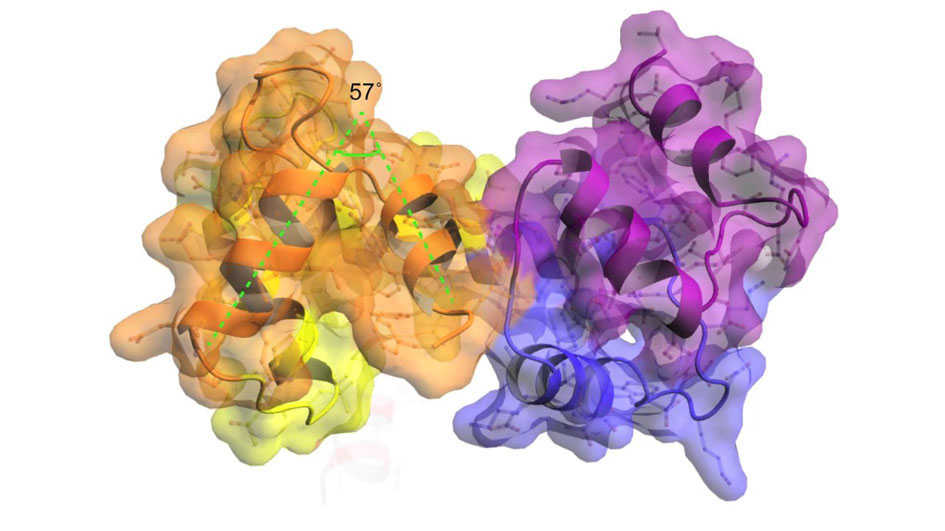

Supplement: Supplementary video 1 — Animation of the calcium-induced changes in HsAPC-1 RD. Morph-model between the calcium-bound HsAPC-1 RD crystal structure and model-2 for the calcium-free HsAPC-1 RD. Structures displayed and coloured as in Fig. 1. [file mmc3.jpg]
